# Leukocyte Count Is Better than LDL-C as Predictor of Novel Carotid Atherosclerosis

**DOI:** 10.3390/biomedicines13081976

**Published:** 2025-08-14

**Authors:** Yan Li, Han Cao, Lei Ding, Xiaotian Shi, Tuge Naren, Qingqing Zhang, Zhong Wang

**Affiliations:** 1 Department of Health Management, Beijing Tsinghua Changgung Hospital, School of Clinical Medicine, Tsinghua University, Beijing 102218, China; 2 Medical Data Science Center, Beijing Tsinghua Changgung Hospital, School of Clinical Medicine, Tsinghua University, Beijing 102218, China; 3 Department of Information Administration, Beijing Tsinghua Changgung Hospital, School of Clinical Medicine, Tsinghua University, Beijing 102218, China; 4 Department of General Medicine, Beijing Tsinghua Changgung Hospital, School of Clinical Medicine, Tsinghua University, Beijing 102218, China

**Keywords:** carotid atherosclerosis, leukocyte count, low-density lipoprotein cholesterol

## Abstract

**Background:** this retrospective cohort study aimed to identify risk factors and establish cutoff values for the initial development of carotid atherosclerosis (CAS) in middle-aged adults. **Methods:** from an initial cohort of 3583 participants, we finally analyzed 1141 individuals. The observation group comprised subjects who developed CAS without concomitant fatty liver disease (FLD), using their last normal clinical measurements as predictors. The control group consisted of participants who remained free of both CAS and FLD throughout the follow-up period. Statistical analyses included Student’s *t*-test, Mann–Whitney U test, and chi-square test for group comparisons, along with logistic regression, COX regression, ROC curve analysis, and Kaplan–Meier survival analysis to identify risk factors and determine optimal cutoff values. Repeated-measures ANOVA assessed longitudinal changes. **Results:** over a mean follow-up of 1.09 years, elevated leukocyte count (AUC: 0.622, 95% CI: 0.540–0.704) and LDL-C (AUC 0.600, 95% CI: 0.516–0.683) were associated with CAS development in middle-aged adults (mean age 49.6 ± 8.0 years). ROC analysis established leukocyte count >5.00 × 10^9^/L and LDL-C >125.1 mg/dL as optimal predictive thresholds. **Conclusions:** leukocyte count and LDL-C are early warning indicators for CAS development within approximately one year, with leukocyte count showing a slightly stronger correlation with arteriosclerosis progression than LDL-C.

## 1. Introduction

Subclinical atherosclerosis in asymptomatic individuals demonstrates a significant independent association with all-cause mortality [1,2,3]. Chronic inflammation and dyslipidemia emerge as established risk factors for acute coronary syndrome [4]. While lifestyle interventions and lipid-lowering therapies can mitigate CAS progression in hyperlipidemic populations [5,6], risk factors for CAS in individuals remain poorly characterized. This study aimed to identify novel predictive biomarkers for early CAS detection to guide timely preventive strategies.

## 2. Materials and Methods

### 2.1. Study Design and Participants

This retrospective analysis enrolled 3583 participants from Beijing Tsinghua Changgung Hospital (2015–2024) with normal baseline carotid and liver ultrasounds. Exclusion criteria included cardiovascular events, pregnancy, and rheumatoid immune diseases [7]. Among these participants, 2761 had undergone at least three carotid artery and liver ultrasound examinations. After excluding those with FLD, a total of 1141 participants completed at least one follow-up examination to assess clinically significant outcomes (presence or absence of CAS), with up to nine follow-up visits conducted to confirm these findings. Access to the data was restricted and was granted under license specifically for this research.

The study was approved by the hospital ethics committee (No. 25295-6-01). Due to the retrospective nature of the study, the ethics committee waived the need of obtaining informed consent. All procedures were performed in accordance with relevant guidelines and regulations.

### 2.2. Clinical and Biological Evaluation

Participants’ age, gender, smoking/drinking status, BMI, blood pressure, and laboratory results (fasting glucose, lipids, leukocyte count, etc.) were recorded. Missing data (<1%) were imputed using adjacent values.

### 2.3. Assessment of CAS

CAS was diagnosed by five trained physicians, each with over 10 years of experience, using Doppler ultrasound systems (Siemens Acuson X700 (Siemens Healthineers, Erlangen, Germany) or Mindray DC-90Pro (Mindray, Shenzhen, China)). The scanning protocol included longitudinal and cross-sectional views, covering the proximal common carotid artery to the distal internal carotid artery bilaterally. CAS was defined by the presence of either carotid intima-media thickening (C-IMT) or carotid atherosclerotic plaque (CAP): C-IMT was diagnosed when the carotid artery intima measured ≥1.0 mm or the bifurcation intima measured ≥1.2 mm. CAP was defined as an intima-media thickness (IMT) ≥1.5 mm that either protruded into the vascular lumen or showed localized thickening exceeding 50% of the adjacent IMT [8,9,10].

### 2.4. Statistical Analysis

Baseline characteristics were compared using *t*-test, Mann–Whitney U tests, or chi-square tests. Logistic regression identified risk factors. ROC curves determined cutoff values. Kaplan–Meier analysis/Cox regression assessed survival rates. Repeated-measures ANOVA evaluated longitudinal changes. A *p*-value less than 0.05 was considered statistically significant.

## 3. Results

### 3.1. Participant Characteristics

As shown in Figure S1, the study included 1141 participants. Among them, 117 individuals (aged 32–85 years at baseline) were classified into the CAS group. No significant differences in clinical characteristics or outcomes were observed between the CAS group and healthy controls(Table 1). As shown in Table 2, the distribution of C-IMT and CAP showed no significant differences between the time of CAS diagnosis and follow-up evaluation (*p* = 0.561). The mean duration from baseline assessment to initial CAS detection was 1.09 years (range: 0.52–3.40 years), with a similar interval of 1.04 years (range: 0.58–4.23 years) observed between initial detection and ultrasound validation.

### 3.2. CAS and Healthy Controls Comparison Before and After PSM for Age and Gender

Compared with healthy controls, participants with CAS were older and more likely to be male. They also exhibited a higher prevalence of smoking and alcohol consumption. Additionally, the CAS group had significantly elevated levels of BMI, systolic and diastolic blood pressure (SBP/DBP), fasting glucose, total cholesterol, triglycerides, LDL-C, ALT, AST, GGT, uric acid, creatinine, leukocyte count, hemoglobin, and platelet count, but lower HDL-C levels (all *p* < 0.05). However, the rates of cardiovascular disease family history did not differ significantly between groups. After PSM for age and gender, CAS patients continued to demonstrate a higher prevalence of elevated levels of LDL-C and leukocyte count (Table 1 and Table S1). Subsequent binary logistic regression analysis confirmed that only LDL-C and leukocyte count remained independently associated with CAS (Table 3).

### 3.3. Cutoff Points of Meaningful Factors with CAS Using ROC Curves and Kaplan–Meier Analysis

**Figure 1:**
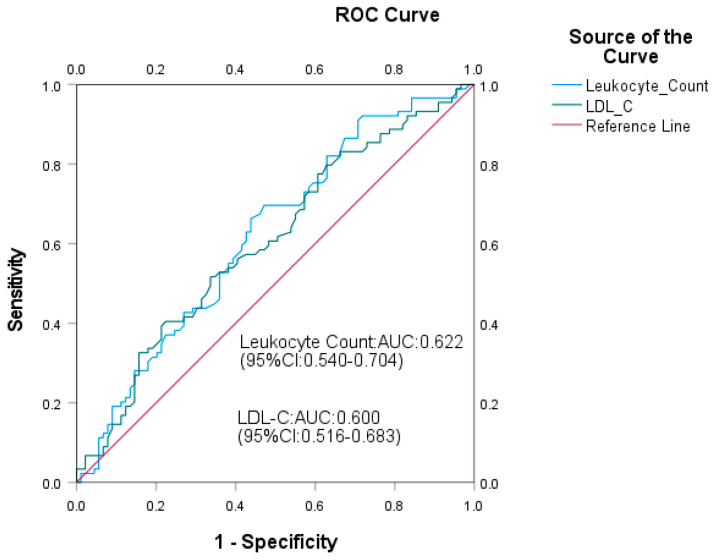
ROC curve analysis of the predictive effect of CAS progression predictors, based on the cutoff values of leukocyte count >5.00 × 10^9^/L and LDL-C > 125.1 mg/dL, respectively.

ROC curve analysis was performed to evaluate the predictive value of significant factors for CAS. The areas under the curves (AUCs) were 0.622 (95% CI: 0.540–0.704, *p* = 0.005) for leukocyte count and 0.600 (95% CI: 0.516–0.683, *p* = 0.022) for LDL-C. No significant differences were observed between the AUCs of leukocyte count and predicted values (*p* = 0.285), LDL-C and predicted values (*p* = 0.285), or between LDL-C and leukocyte count (*p* = 0.291) (Figure 1). Optimal cutoff values were established at 125.1 mg/dL for LDL-C and 5.00 × 10^9^/L for leukocyte count, with values above these thresholds classified as high-level groups.

**Figure 2:**
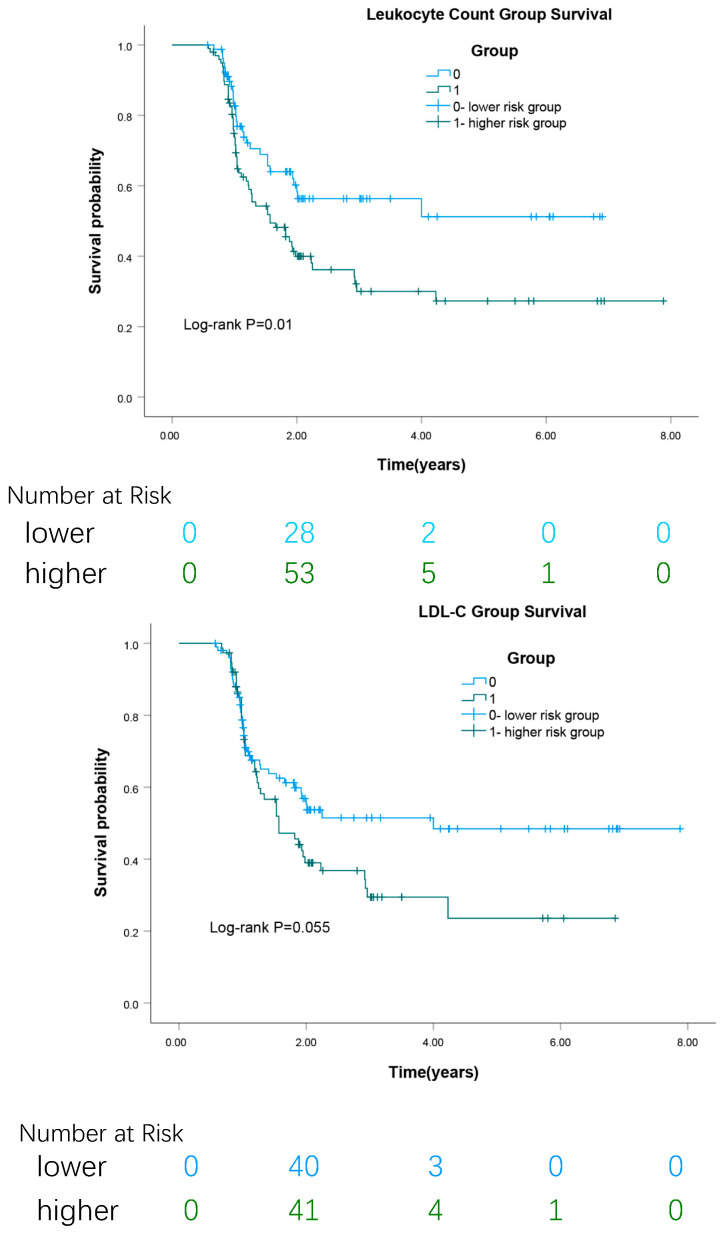
Kaplan–Meier analysis the survival rates, based on the classification of cutoff values of leukocyte count and LDL-C different levels, respectively.

Kaplan–Meier analysis (Figure 2) revealed that the high leukocyte count group showed significant association with CAS progression (log-rank *p* = 0.01), while LDL-C groups demonstrated borderline significance (log-rank *p* = 0.055). Stratification analysis showed the following: (1) For leukocyte count, 60.2% of high-risk and 37.5% of low-risk individuals developed CAS. (2) For LDL-C, 60.5% of high-risk and 42.2% of low-risk individuals developed CAS. The high leukocyte count group exhibited significantly increased risk of CAS progression (HR 1.766, 95% CI 1.138–2.743), as did the high LDL-C group (HR 1.570, 95% CI 1.056–2.084). Multivariable analysis (Table 4) confirmed these associations.

The leukocyte subclass Cox model (Table S2) significantly improved upon the null model, with two key predictors: (1) Neutrophils demonstrated a dose-dependent relationship (aHR 1.29 per 10^9^/L increase, 95% CI 1.01–1.64; *p* = 0.043). (2) Eosinophils showed the strongest association (aHR 7.47 per 10^9^/L, 95% CI 1.36–40.94; *p* = 0.021), suggesting clinically meaningful risk even at minimal elevations.

### 3.4. Longitudinal Changes in Leukocyte Count

Elevated leukocyte count was observed exclusively prior to CAS diagnosis. However, no significant intergroup differences were observed at subsequent time points (Table S3, Figure S2). Leukocyte count decreased post-CAS diagnosis (baseline: 5.56 ± 1.22; follow-up: 5.35 ± 1.31, *p* = 0.037), suggesting its role as an early warning marker.

## 4. Discussion

In this retrospective cohort study of 1141 Chinese adults evaluated by carotid ultrasound, we identified elevated leukocyte counts and LDL-C levels in CAS patients prior to disease onset. Notably, both biomarkers demonstrated predictive value for CAS, with leukocyte count showing superior predictive performance after adjustment for age and gender (AUC 0.622 vs. 0.600 for LDL-C). Our findings revealed that both C-IMT and CAP can manifest as initial presentations in newly diagnosed CAS cases. Importantly, longitudinal follow-up (median 1.04 years, range 0.58–4.23 years) showed no evidence of progression from C-IMT to CAP, suggesting that these may represent distinct phenotypic manifestations rather than sequential stages of carotid atherosclerosis.

### 4.1. The Significance of CAS

Previous studies have established associations between leukocytosis and both FLD [11] and immune disorders [7,12]. To minimize confounding effects, we excluded participants with FLD (confirmed by ultrasound) and those reporting immune diseases (via standardized questionnaire). Our findings align with existing studies in the literature showing peak CAS progression in adults aged 50–59 years [13], as our PSM CAS cohort had a mean age of 49.6 years [14]. Current evidence suggests that while C-IMT and CAP serve as markers of CAS, they may not fully reflect disease severity, with mixed or soft plaques frequently observed even in individuals with normal C-IMT [15]. Our longitudinal data extend these observations by demonstrating stable lesion characteristics during follow-up, with neither isolated C-IMT nor CAP showing significant progression over the median 1.04-year observation period. Importantly, concurrent C-IMT and CAP lesions remained similarly stable, and newly developed CAS lesions exhibited no distinctive morphological patterns. These findings collectively suggest that early-stage CAS may follow an indolent course, with lesion characteristics remaining relatively stable in the short-to-medium term.

### 4.2. Association Between Leukocyte Counts, LDL-C, and CAS

Leukocyte count serves as a key inflammatory marker that participates in both the initiation and progression of atherosclerosis [16,17]. Our findings align with existing evidence showing significant associations between leukocyte count and lipid profiles (HDL-C, LDL-C, TG, and TC) [18,19]. The potential mechanism may involve VLDL receptor (VLDLR) mRNA expression in peripheral leukocytes, which appears to contribute to CAS development in healthy individuals, particularly in patients exhibiting low VLDL-C but high VLDLR mRNA expression [20]. This process may be mediated through VLDLR-fibrin βN-domain (β40-66) interactions that promote endothelial permeability and leukocyte transmigration [21], a pathway that can be inhibited by β15-42 interactions with endothelial receptors [22] or through anti-VLDLR monoclonal antibodies (1H10, 1H5) [23].

Neutrophil count serves as an independent predictor of all-cause and cardiovascular mortality in neurologically asymptomatic carotid stenosis patients [24], while eosinophil-specific LNK (SH2B3) deficiency promotes both eosinophilia and arterial thrombosis [25]. Although monocytes may drive CAP development without significantly affecting carotid C-IMT [26], our findings demonstrate that early CAS progression is predominantly associated with acute inflammatory responses mediated by neutrophils and eosinophils, with no observed transition from C-IMT to CAP during short-term follow-up. Conversely, monocytes appear to orchestrate the chronic phase of lesion progression toward advanced CAP. These observations collectively suggest the leukocyte subtypes in CAS pathogenesis—with neutrophils and eosinophils potentially initiating early pathological changes, while monocytes may orchestrate subsequent arterial remodeling.

This discrepancy may reflect population differences, as our PSM cohort primarily comprised apparently healthy middle-aged women with low LDL-C levels. Notably, our data confirm that elevated leukocyte count occur specifically before CAS diagnosis but not thereafter, reinforcing its potential as an early warning indicator. These findings contrast with reports of faster arterial deterioration in young Caucasian males, where atherosclerosis typically progresses from the abdominal aorta to other vascular beds [27], and highlight the importance of sex-specific interactions between leukocyte count and plaque stability [28].

These results underscore the clinical importance of monitoring leukocyte count as part of early CAS risk assessment, particularly in middle-aged female populations without conventional lipid risk factors. The findings advocate for enhanced clinical vigilance and targeted early interventions in these demographic groups.

### 4.3. Strengths and Limitations

To our knowledge, this represents the first study to identify early warning indicators for CAS and characterize their temporal relationship with disease onset. However, several limitations should be acknowledged. First, while we cross-validated available electronic medical records, the majority of participants’ medical and family histories relied on self-reporting, which may introduce recall bias. Second, our cohort consisted predominantly of Han Chinese (over 50% female), potentially limiting the generalizability of our findings to other ethnic and gender groups. Third, the regional confinement of our study population may further restrict the external validity of our results. Existing evidence underscores the pivotal role of immune–inflammatory mechanisms in arteriosclerosis pathogenesis, particularly through systemic inflammation [29], monocyte–macrophage activity [30], and coagulation–fibrinolysis imbalances [31]. Unfortunately, our study was constrained by the available clinical data, with limited inflammatory markers collected during follow-up. While these limitations were inherent to our study design, we intend to address them in future multicenter collaborative studies to validate our findings.

## 5. Conclusions

Elevated leukocyte count (>5.00 × 10^9^/L) and LDL-C (>125.1 mg/dL) serve as clinically practical early warning indicators for CAS development. The strong association between leukocytosis and CAS progression particularly underscores the potential involvement of immune–inflammatory mechanisms in the pathogenesis of arteriosclerosis, a finding that merits further mechanistic investigation.

## Figures and Tables

**Table 1:** Baseline characteristics among healthy controls and the CAS group.

	Control n = 1024	CAS n = 117	*p*-Value
Age, years	36.4 ± 7.9	52.6 ± 10.0	<0.001
Female, (%)	729 (71.2)	73 (62.4)	<0.001
Alcohol status, %	94 (9.2)	19 (16.2)	<0.001
Smoking status (former or active), %	49 (4.8)	9 (7.7)	<0.001
Family history of cardiovascular disease, %	97 (9.5)	20 (17.1)	0.064
Time interval, years	1.06 (0.98, 1.29)	1.09 (0.99, 1.84)	0.076
Type2 diabetes	1	1	
Hypertension	0	1	
BMI, kg/m^2^	21.8 ± 2.5	22.8 ± 2.3	<0.001
SBP, mmHg	114.9 ± 13.2	123.6 ± 15.8	<0.001
DBP, mmHg	69.8 ± 9.5	73.9 ± 10.2	<0.001
MAP, mmHg	84.0 (77.7, 90.3)	90.0 (82.2, 99.2)	<0.001
Fasting glucose, mg/dl	86.0 ± 8.5	91.4 ± 0.72	<0.001
Total cholesterol, mg/dl	182.2 ± 32.8	197.6 ± 33.6	<0.001
Triglycerides, mg/dl	73.5 (57.5, 97.4)	91.2 (73.5, 129.2)	<0.001
LDL cholesterol, mg/dl	108.5 ± 29.3	124.7 ± 29.7	<0.001
HDL cholesterol, mg/dl	57.1 ± 13.1	54.0 ± 12.0	<0.001
ALT, U/L	14.7 (11.2, 19.4)	18.2 (13.7, 22.9)	<0.001
AST, U/L	18.4 (15.9, 21.6)	20.1 (18.0, 24.4)	<0.001
GGT, U/L	12.7 (9.3, 17.3)	16.8 (12.5, 22.0)	<0.001
Uric acid, mg/dl	5.03 ± 1.28	5.27 ± 1.11	<0.001
Creatinine, mg/dl	0.68 ± 0.13	0.70 ± 0.15	<0.001
Hemoglobin, g/L	138.3 ± 16.0	141.3 ± 15.1	<0.001
Platelet count, 10^9^/L	248.6 ± 54.0	232.6 ± 57.1	0.002
Leukocyte count, 10^9^/L	5.55 ± 1.23	5.56 ± 1.22	<0.001

**Table 2:** Characteristics of CAS participants’ ultrasound results before and after propensity score matching for age and sex.

	Before PS Matching				After PS Matching			
	C-IMT	CAP	C-IMT+CAP	Time intervals, y	C-IMT	CAP	C-IMT+CAP	Time intervals, y
**Baseline**	0	0	0		0	0	0	
**CAS diagnosis**	49(41.9%)	46(39.3%)	22(18.8%)	1.09(0.99,1.84)	38(42.7%)	44(49.4%)	7(7.9%)	1.03(0.91,1.53)
**Follow-up**	44(37.6%)	49(41.9%)	24(20.5%)	1.04(0.92,1.53)	36(40.4%)	39(43.8%)	14(15.7%)	1.04(0.93,1.53)

**Table 3:** Factors associated with CAS using binary logistic regression.

	Univariate			Multivariate		
	OR	95% CI	*p*-value	AOR	95% CI	*p*-value
SBP	1.014	1.002–1.026	0.022	1.011	0.999–1.024	0.084
LDL-c	1.327	1.032–1.706	0.028	1.368	1.062–1.763	0.015
Leukocyte count	1.186	1.029–1.367	0.019	1.172	1.006–1.366	0.042

**Table 4:** Multivariable Cox regression results for CAS.

	Unit	aHR (95% CI)	Wald χ^2^	*p*-Value
Leukocyte Count	per 10^9^/L	1.21 (1.04–1.40)	6.28	0.012
LDL-C	per 1 mmol/L	1.37 (1.06–1.77)	5.97	0.015

## Data Availability

The data that support the findings of this study are available from Beijing Tsinghua Changgung Hospital, School of Clinical Medicine, Tsinghua University, but restrictions apply to the availability of these data, which were used under license for the current study, and so are not publicly available.

## Supplementary Materials

The following supporting information can be downloaded at: https://www.mdpi.com/article/10.3390/biomedicines13081976/s1, Table S1: Baseline characteristics between healthy controls and CAS group after PSM for age and gender; Table S2 Leukocyte subclass mul-tivariable Cox Regression results for CAS; Table S3 Leukocyte count comparisons between and within groups after propensity score matching for age and gender. Figure S1. Study flowchart depicted total number of subjects enrolled and reasons for exclusion; Figure S2. Leukocyte count differences of within CAS group and between groups at three time points.
