# Supplementary material for: Leukocyte Count Is Better than LDL-C as Predictor of Novel Carotid Atherosclerosis
Source: Biomedicines. 2025 Aug 14;13(8):1976. doi: 10.3390/biomedicines13081976 (PMC12383423; doi:10.3390/biomedicines13081976)
Supplement: Supplementary file 1 [file biomedicines-13-01976-s001.zip › Supplemental Figures.pdf]

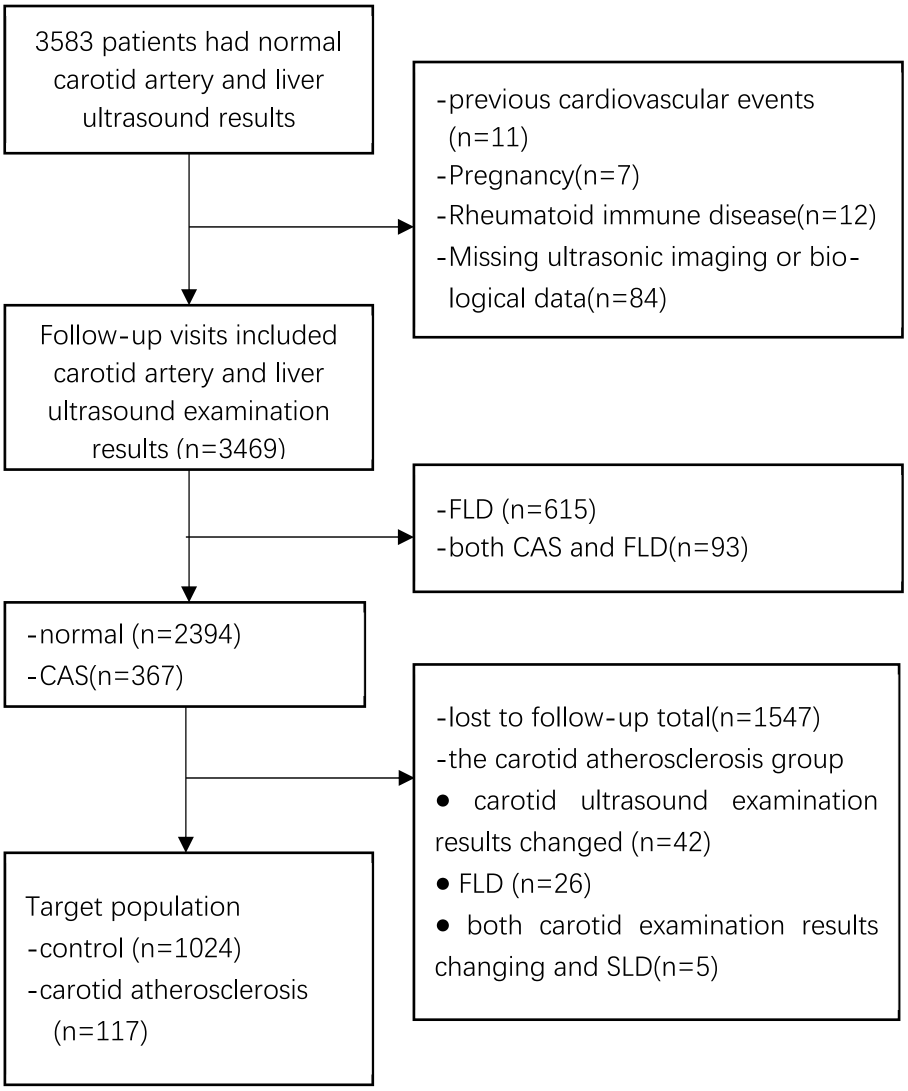

Figure S1. Study flowchart depicted total number of subjects enrolled and reasons for exclusion

CAS=carotid atherosclerosis FLD= fatty liver disease

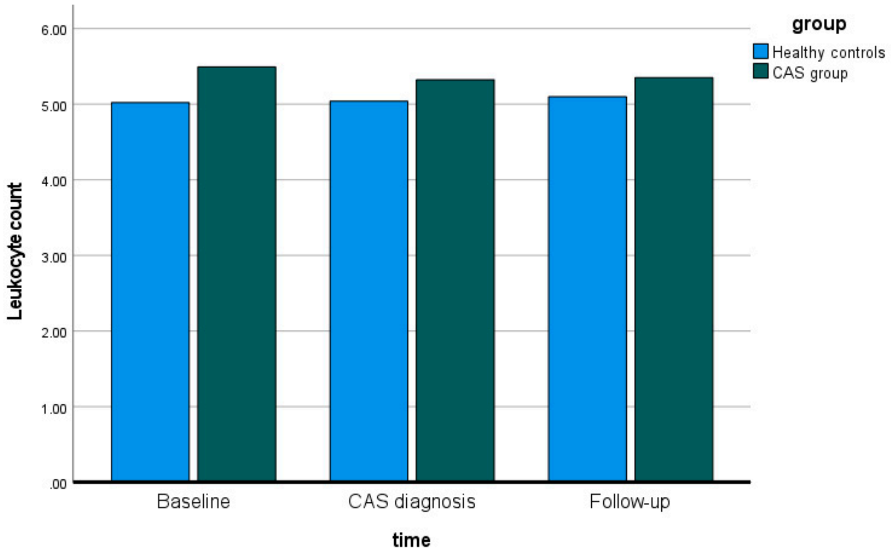

Figure S2. Leukocyte count differences of within CAS group and between groups at three time points.
