# Supplementary material for: Leukocyte Count Is Better than LDL-C as Predictor of Novel Carotid Atherosclerosis
Source: Biomedicines. 2025 Aug 14;13(8):1976. doi: 10.3390/biomedicines13081976 (PMC12383423; doi:10.3390/biomedicines13081976)
Supplement: Supplementary file 1 [file biomedicines-13-01976-s001.zip › Supplemental Tables .pdf]

Table S1. Baseline characteristics between healthy controls and CAS group after PSM for age and gender.

	Control n=89	CAS n=89	P value
Age,years	49.5±8.2	49.6±8.0	0.940
Female,N(%)	62(69.7)	58(65,2)	0.522
Alcohol status,%	12(13.5)	16(18.2)	0.392
Smoking status(former or active),%	9(10.1)	6(6.8)	0.431
Family history of Cardiovascular disease,%	13(14.6)	15(17)	0.657
Time interval,years	1.03(0.97,1.27)	1.03(0.91,1.53)	0.567
Type2 diabetes	0	0	
Hypertension	0	0	
**Physical examination**			
BMI,kg/m^2	22.6±2.3	22.8±2.3	0.708
SBP, mmHg	117.0±17.1	121.3±15.2	0.081
DBP, mmHg	71.7±12.0	73.5±10.4	0.281
MAP,mmHg	86.8±12.7	89.4±11.2	0.145
**Lab test**			
Fasting glucose, mg/dl	88.6±14.4	90±11.3	0.517
Total cholesterol, mg/dl	188.4±32.4	195.3±34.4	0.167
Triglycerides , mg/dl	89.4(69.0,113.3)	89.4(69.0,129.2)	0.638
LDL cholesterol , mg/dl	111.2±31.3	122.7±30.9	0.019
HDL cholesterol, mg/dl	57.1±13.5	54.0±12.0	0.100
ALT,U/L	17.4(14.6,21.0)	18.2(13.8,22.9)	0.869
AST, U/L	20.8(18.4,23.3)	20.3(18.0,24.3)	0.791
GGT,U/L	15.3(11.4,21.5)	16.6(12.2,22.3)	0.290
Uric acid, mg/dl	4.69(3.91,5.92)	5.09(4.30,6.02)	0.115
Creatinine, mg/dl	0.70±0.14	0.69±0.14	0.627
Hemoglobin, g/L	137.4±18.1	141.5±16.1	0.113
Platelet count,10^9/L	235.0±45.5	237.3±58.8	0.778
Leukocyte count, 10^9/L	5.02±1.33	5.49±1.21	0.015

Table S2 Leukocyte subclass multivariable Cox Regression results for CAS

	β	SE	Wald	P value	aHR (95% CI)
Neutrophils	0.251	0.124	4.10	0.043	1.29 (1.01-1.64)
Lymphocytes	0.351	0.221	2.52	0.112	1.42 (0.92-2.19)
Monocytes	-0.434	1.131	0.15	0.701	0.65 (0.07-5.95)
Eosinophils	2.010	0.868	5.36	0.021	7.47 (1.36-40.94)
Basophils	-13.105	7.937	2.73	0.099	<0.01 (<0.01-11.61)

Table S3 Leukocyte count comparisons between and within groups after propensity score matching for age and gender

	Control	CAS	F	P
Baseline	5.02±1.33	5.49±1.21	6.086	0.015
CAS diagnosis	5.04±1.26	5.32±1.27	2.243	0.136
Follow-up	5.10±1.50	5.35±1.27	1.454	0.230
F	0.281	1.939		
P value	0.755	0.147		
Group（F,P）	3.562	0.061		
Time（F,P）	0.663	0.517		
Time*Group（F,P）	1.557	0.214		
